# Supplementary material for: Coach–Athlete Relationships and Mental Health: An Exploratory Study on Former Female NCAA Student-Athletes
Source: Int J Environ Res Public Health. 2025 Oct 30;22(11):1652. doi: 10.3390/ijerph22111652 (PMC12652550; doi:10.3390/ijerph22111652)
Supplement: Supplementary file 1 [file ijerph-22-01652-s001.zip › ijerph-3846135-supplementary.pdf]

*Article*

# Coach-Athlete Relationships and Mental Health: A*n Exploratory Study on Former Female NCAA Student-Athletes*

**Ashley R. Kernan [1], Michael R. Cope [1,*], Jonathan A. Jarvis [1] and Mikaela J. Dufur [1]**

## Supplementary materials

Table S1 provides the themes we developed based on prior research on coach–athlete relationships, athlete well-being, and gendered experiences in collegiate sport. Questions addressed topics such as recruitment experiences and explored early socialization to address transitions from youth to collegiate athletic programs. The questions were also designed to focus on perceptions of support and pressure, injury and recovery, mental health, and reflections on quality of life. Finally, we wanted to examine communication between coach and student-athletes, and the development of the relationships that student-athletes develop with their coaches, highlighting long-term implications of collegiate sport participation.

**Table S1.** Summary of Interview Guide.

| Thematic Section | Example Questions | Example Supporting Literature |
|---|---|---|
| 1. Background and Introduction | • Please tell me about yourself — name, age, university, sport, and how long you've played.<br>• How did you start doing this sport? Describe that experience. | [35] |
| 2. Transitioning to Collegiate Athletics | • How was college sports different from earlier experiences?<br>• Walk me through your recruiting experiences with universities and coaches.<br>• How was your experience with the school you ultimately attended? | [18-20,47] |
| 3. Relationships with Coaches | • Describe your interactions with coaches during recruiting.<br>• How did those interactions differ once you were on the team? | [55,56] |

| | | |
|---|---|---|
| | • What was different? Can you give an example? | |
| 4. Daily Life and Routine | • What was/is your daily routine like between classes and your sport? Did it change over time? | [4, 6] |
| 5. Interpersonal Dynamics and Team Atmosphere | • Describe your personal experiences with coaches and how they affected you. • Explain the overall team atmosphere with the coaches — did it change over time? | [31,46] |
| 6. Positive and Negative Experiences | • If experiences were negative, expand on why — any yelling, punishment, frequency, examples? • If experiences were positive, expand on why — what made them positive, frequency, examples? • How did these experiences affect you? Did they bother you? • Was there a shift from positive to negative (or vice versa)? Describe that transition. • When during your collegiate career did these experiences occur (first year, last year, etc.)? • How have these interactions affected your daily life — for better or worse? Why? | [3,25-27] |
| 7. Long-Term Reflection | • Looking back, how have these experiences influenced your view of sport, success, or relationships? • How do you think these experiences will shape your life after college athletics? | [6, 8,40,41] |

**Disclaimer/Publisher's Note:** The statements, opinions and data contained in all publications are solely those of the individual author(s) and contributor(s) and not of MDPI and/or the editor(s). MDPI and/or the editor(s) disclaim responsibility for any injury to people or property resulting from any ideas, methods, instructions or products referred to in the content.
